# Interactive effects of visuomotor perturbation and an afternoon nap on performance and the flow experience

**DOI:** 10.1371/journal.pone.0171907

**Published:** 2017-02-09

**Authors:** Kosuke Kaida, Yoshihiro Itaguchi, Sunao Iwaki

**Affiliations:** 1 Institute for Information Technology and Human Factors, National Institute of Advanced Industrial Science and Technology (AIST), Tsukuba, Ibaraki, Japan; 2 Department of System Design Engineering, Keio University, Yokohama, Kanagawa, Japan; Waseda University, JAPAN

## Abstract

The present study was designed (1) to clarify the relationship between the flow experience and improvements in visuomotor skills, (2) to examine the effects of rotating the axis of a computer mouse on visuomotor skills, and (3) to investigate the effects of sleep for improving visuomotor skills. Participants (N = 18) responded to Perturbation and nap (PER+Nap), No-perturbation and nap (NoPER+Nap) and Perturbation and rest (PER+Rest) conditions. In the PER+Nap condition, participants conducted a visuomotor tracking task using a computer mouse, which was accompanied by perturbation caused by rotating the axis of their mouse. After the task, they took a 90 min nap. In NoPER+Nap condition, they conducted the same visuomotor task without any perturbation and took a nap. In the PER+Rest condition, participants conducted the task with the perturbation and took a 90 min break spent reading magazines instead of taking a nap. Results indicated (1) the flow experience did not occur when participants’ skills and the degree of the visuomotor challenge were matching, (2) improvements of visuomotor skills occurred regardless of the perturbation, (3) improvements of visuomotor skills occurred unrelated to the flow experience, or to mood states, and (4) improvements of visuomotor performance occurred regardless of sleep. These findings suggest that improvements of visuomotor skills occur regardless of mood status and occur independently of perturbations by axis rotation. The study also suggests that the acquisition of skills is related to merely the time elapsed since learning, rather than to sleep.

## Introduction

People find activities they are engaging in to be interesting when they are dedicated to an activity that is moderately difficult. According to the flow theory proposed by Chiksentmihaliy [1, 2], task difficulty in relations to an individual’s skills affect the feelings of enjoyment in doing a task. Moreover, a sense of enjoyment is experienced when there is a perfect balance between task difficulty and the performer’s skills. Then, individuals can feel a deep involvement with the task and they might feel they merge with the task [3]. People also feel that the situation is smooth and flowing and forget the passage of time. Individuals often report positive feelings when engaging in such tasks, which has been named “the flow” experience [1, 2].

Individuals believe that they are performing a given task well when they are experiencing the flow because their mental and physical conditions are believed to be at their near best [1]. People devote themselves to doing the task and modify their skills to improve their performance [4], and therefore, theoretically, their ability to execute the task could be improved by the flow experience. This could happen when perceived task difficulties (i.e., challenges) and their abilities perfectly match each other. Contrary to this, when a task is too easy (i.e., the diffeculty level is too low), individuals might easily get bored, which could result in irritation and tiredness, causing distractions. On the other hand, if the task difficulty were perceived as being too high compared to the performer’s skills (i.e., the challenge level is too high), he or she might feel negative feelings, including anxiety. As a result, if the challenge level were too high or too low compared to a performer’s skills, the flow state might not be facilitated. Therefore, adjusting the task difficulty to correspond to the performer’s skill level is critically important for inducing the flow [1, 2].

Despite its positive attributes, however, the flow theory has not been well substantiated from the perspective of behavioral science. Practitioners have attempted to apply this theory to educational [5] and sports settings [6–8]. However, these attempts have at times been pointless, because the types of abilities and how these abilities are enhanced through the flow experience were not clarified. Examining the flow theory through behavioral experiments under controlled settings is necessary before the theory can be accurately and efficiently applied to practical situations. The aim of the present study was to clarify the relationship between the flow and skills learning.

We selected a visuomotor adaptation task to examine the flow theory [9–11]. The task consisted of skills that are fundamental to our daily life. In the visuomotor adaptation task that was used in this study, participants followed a moving dot on a PC screen by using a mouse pointer (no-rotation condition). This is a task that is similar to web-browsing that people do on a daily basis. In the axis rotation condition, however, the axis of the mouse point was rotated between 0 and 120 degrees in a clockwise direction to cause a perturbation and distract participants from following the moving dot by deviating the direction of mouse movements from the expected axis. After repeated practice, participants do acquire the skill of correctly manipulating a mouse with a rotated axis direction by gradually developing their skill to fit the new environment (i.e., rotated axis). This is a typical implicit learning phenomenon, which is known as “visuomotor adaptation” [12].

To compare the effects of visuomotor perturbation on visuomotor skills, performance in the axis rotation condition was compared with that of the no-rotation condition before and after practicing the task. In the visuomotor task, participants were requested to follow the moving dot shown on a LCD screen with their dominant hand by using the mouse pointer, which is a skill that is known to improve with practice [10]. It was assumed that using the mouse with its axis-rotated would deteriorate the performance.

The degree of difficulty involved in using a PC mouse with its axis rotated can be easily and accurately manipulated by increasing the degree of rotation; such that higher the degree of rotation, the more difficult would be the task. This feature of axis rotation is ideal for examining the flow theory. According to the flow theory, the flow would be experienced even when conducting a simple task, as long as the task is matched with the skill level of the performer [1]. An individual would experience the flow whenever he or she attempt to close the gap between intrinsic skills and increased task difficulty.

An additional aim of the present study was to investigate the effect of sleep on learning visuomotor skills. Certain previous studies have reported that skill acquisition in implicit motor learning tasks, such as imagery learning [13] and juggling [14], occurs during sleep [15–20]. However, other studies have failed to demonstrate this effect in adults [21–25], or children [26, 27]. We assumed that emotional states such as the flow, which is experienced during sports practice might explain the controversial findings regarding the nap effect on visuomotor skills. This assumption is reasonable when considering relevant evidence from previous studies suggesting that emotional states during memory encoding might play an important role in memory consolidation during subsequent sleep [28, 29]. Similarly, it was expected that the flow, which is a positive emotional state experienced during task performance, could enhance visuomotor skills during subsequent sleep. In addition, this study was designed to investigate the relationship between the flow experience and acquisition of visuomotor skills during sleep (i.e., a 90 min afternoon nap).

The hypotheses of the present study were: (1) matching own skills to a given difficulty in a visuomotor task would induce the flow experience compared to the non-matching condition in which the task difficulty was constant, (2) flow experience during the task would promote acquisition of visuomotor adaptation skills, and (3) visuomotor adaptation skills that are enhanced by the flow experience would improve during sleep more than in the no-sleep condition.

## Methods

### Participants

Participants were 9 men and 9 women aged 18–35 years (mean = 26.2 years, *SD* = 5.55). A standardized interview conducted before the experiment confirmed that all the participants were right-handed, they had no current physical or mental health problems, they did not suffer from any sleep disturbances, they were not currently using any medication, they were non-smokers, and they had not engaged in shift work or traveled to a different time zones within the previous three months. Participants also reported that they have natural or corrected visual accuracy of over 0.8 (18/20 vision). Participants were asked to abstain from food and beverages containing caffeine or alcohol after 18:00 h on the day prior to the experiment and throughout the experimental period. The ethical considerations of the experimental protocol were reviewed and approved by the review board at the National Institute of Advanced Industrial Science and Technology (AIST) of Japan, according to the principles expressed in the Declaration of Helsinki. All participants had given prior written informed consent for participating in the study.

### Procedure

Each participant was tested under three experimental conditions: (1) perturbation and nap (PER+Nap), (2) no-perturbation and nap (NoPER+Nap), and (3) perturbation and rest (PER+Rest). In the PER+Nap condition, participants conducted the visuomotor task with perturbation caused by the axis rotation of the mouse. After the task, participants took a 90 min nap. In the NoPER+Nap condition, participants conducted the visuomotor task without perturbation and then took a nap. In the PER+Rest condition, they conducted the perturbation task and instead of a nap they took a 90 min break which was spent reading the National Geographic magazines.

Participants slept in the laboratory on one day to familiarize themselves with the laboratory environment, in order to avoid the first-day effect. The procedure of the experiment was explained on this day and participants practiced performing the tasks and responding to questionnaires. One week later, they performed one of the three experimental conditions. Days between conditions were separated by at least seven days to avoid any carryover effects from the previous experiment. Moreover, any carryover effects of the tasks were controlled by counterbalancing the order of conditions among participants.

**Fig 1:**
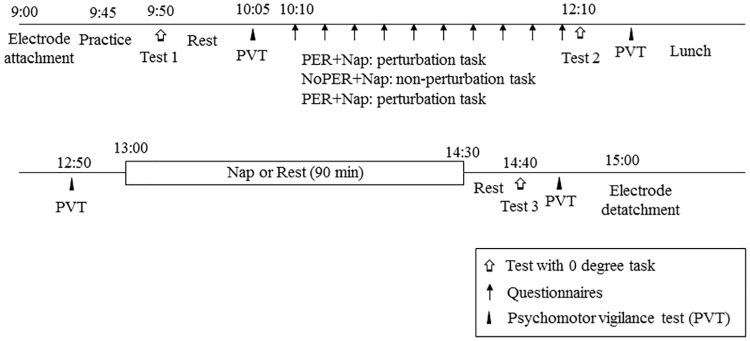
Design of the experiment. Three experimental conditions were carried out: (1) perturbation and nap (PER+Nap), (2) no-perturbation and nap (NoPER+Nap), and (3) perturbation and rest (PER+Rest). In the PER+Nap condition, participants conducted the visuomotor task with perturbation caused by the axis rotation of the mouse. After the task, participants took a 90 min nap. In the NoPER+Nap condition, participants conducted the visuomotor task without perturbation and then took a nap. In the PER+Rest condition, they conducted the perturbation task and instead of a nap they took a 90 min break.

Participants arrived in the laboratory at 9:00 on the day of the experiment as shown in Fig 1. After that, the electrodes were attached for polysomnography. At approximately 9:50, the first session (Test 1) of the visuomotor task (with no-mouse axis rotation, i.e., 0 degrees) was conducted to assess their baseline visuomotor skills which took approximately two min. At 10:00, the 5 min psychomotor vigilance task (PVT) was conducted to assess their vigilance level. Then, approximately at 10:20 the main task started, which included 10 consequent 8 min sessions, each of which consisted of 4 trials (i.e., total 40 trials) with one trial consisting of four epochs of 30 sec. After each session, participants responded to a questionnaire for approximately one min. Participants were allowed to take short breaks between trials when executing the main task. The total duration of the main task varied among participants due to different times spent on short breaks, although it was no longer than 100 min. The second test session (Test 2) of visuomotor skills was conducted at approximately 12:00 noon, followed by the PVT at approximately 12:10. The purpose of the second test with no axis rotation was to assess the effects of practice or perturbation on the visuomotor skill, which was assigned to all three conditions to assess differences in skills learning compared to the baseline (i.e., Test 1). After a lunch break (12:20–13:00), participants took a nap or rested in the sound insulated laboratory from approximately 13:00 to 14:30 (i.e., 90 min) with the electrodes mounted. At approximately 14:30, participants took a 10 min break for using the restroom and for light exercise to reduce sleep inertia. The third test session (Test 3) was started at 14:40 and all experimental procedures were completed when it was completed. Test 3 was conducted to assess the effect of taking a nap on skills acquisition. The task had no axis rotation.

### Performance task

#### Visuomotor task

In the visuomotor task, participants were requested to follow the moving dot shown on a LCD screen (diameter: 5 mm, velocity: 14.36 cm/s; range of X and Y axis: -100–100 pixels) with their dominant hand by using the mouse pointer. The mouse axis was rotated in 14-degree increments to make the task progressively more difficult if the mean distance between the moving dot and the mouse cursor improved by 3% to 20% in perturbation conditions (PER+Rest and PER+Nap) compared to the previous trial. Moreover, axis rotation was returned closer to the 0 position in 7-degree increments to make the task easier if the performance deteriorated by 50% to 90% compared to the previous trial. Axis rotation was conducted cumulatively throughout the ten sessions whereas rotation per trial was conducted only once, such that the mean axis rotation was 63 degrees (range 0–112 degrees). Feedback on the degree of axis rotation (i.e., challenge level) was given to each participant before a trial by showing the information on the screen. It was assumed that task difficulty in the perturbation condition (PER+Rest and PER+Nap conditions) would constantly be maintained at a level optimal to the skill level of each participant by using the above procedure. It was theoretically assumed that this setting in which an individual’s skill level and task difficulty were matching would enhance the flow experience when performing the task. Task difficulty in the no-perturbation conditions (NoPER+Nap condition) was set to the minimal (i.e., no axis rotation) throughout the experiment. Visuomotor performance was determined by calculating the distances between the target and the point where the mouse cursor was located by using pixels. All stimuli were displayed on a 23-inch LCD monitor with a 1920 × 1080 resolution (HP Elite Display E231) connected to a computer. The participants observed the stimuli at a distance of 57 cm. A program written in MATLAB controlled the experimental schedule, using the Psychophysics Toolbox extensions [30, 31].

#### Psychomotor vigilance test

The PVT [32] was used to assess the degree of vigilance in participants. The PVT is a simple visual reaction time (RT) test that requires a participant to respond as fast as possible (by using a key press) to a red target (a red number indicating the time in ms) appearing in the center of a screen. Interstimulus intervals varied between 2–10 s and participants performed the task for 5 minutes per session. Median RT was calculated for each participant and condition.

### Questionnaire

#### Flow checklist

The Flow Checklist (FCL), which was originally developed in Japanese by Ishimura (2008) [33], was used to assess the flow state. The FCL consists of 10 items that are rated by using a 7-point Likert scale ranging from 1 (*does not apply at all*) to 7 (*highly applicable*). FCL items are categorized into three independent factors: “Confidence in competence,” “Rising to the challenge,” and “Positive emotions and immersion,” with each factor consisting of 2–4 items. Items in Factor 1 (Confidence) include, “Everything is going well,” “I am able to control situations,” “I am confident in managing matters,” and “I am in control of my behavior/movements.” Items in Factor 2 (Challenge) include, “I feel my work is challenging” and “I am making progress toward reaching my goals.” Items in Factor 3 (Immersion) include, “I feel time flies,” “I am in a state of complete concentration,” “I am completely immersed,” and “I am enjoying my work.” The reliability of the FCL with three factors has been confirmed in a previous study using factor analysis that demonstrated adequate Cronbach’s alpha coefficients [33]. Moreover, the reliability of the FCL in the present study was confirmed by adequate Cronbach’s alpha (α = 0.98 for Confidence; α = 0.98 for Challenge; α = 0.87 for Immersion). FCL was used to assess the flow experience during the main task of this study. Participants were asked to circle a number indicative of their current feelings after each session of the main task using the 7-point scale. Mean scores for each factor were calculated for each participant and condition. The scores obtained from the first and last sessions were omitted to avoid potential biases.

#### Mood status

Visual analogue scales (VAS) were used to assess participant’s mood during the main task on dimensions of “anxious,” “sleepy,” “fatigued,” “apathetic/vigorous,” “confused,” “angry,” and “sad.” Participants drew a line on the 100 mm scale to indicate their current moods after each session. Mean scores were calculated for each participant for each mood item. The scores obtained from the first and last sessions were omitted to avoid potential biases.

### Polysomnography

The EEG (at Cz referenced to linked electrodes at the earlobes), the electrooculogram (EOG, from electrodes at the outer canthi) and the electromyogram (EMG, from electrodes at the chin) were recorded for standard polysomnography. The sampling rate of all signals was 1000 Hz (24-bit AD conversion) with time constants of 0.3 s for the EEG, 3.2 s for the EOG, and 0.03 s for the EMG. Electrode impedance was maintained below 10kΩ. Electrophysiological data were recorded with a portable digital recorder (PolymateV AP5148, Miyuki Giken Co., Ltd, Japan).

Sleep architecture was determined according to standard criteria [34, 35] using the EEG recordings at Cz for succeeding 30-sec epochs. Total sleep time and time spent in the different sleep stages (wake—W, sleep stages 1, 2, 3, 4 – S1-S4; slow wave sleep—SWS, sum of S3 and S4, REM sleep) was calculated in min for each day.

### Statistical analysis

Recognition performance was analyzed by a two-way Condition (3) × Time (3) analysis of variance (ANOVA). To control for the Type 1 error associated with violation of the sphericity assumption, degrees of freedom greater than one were reduced by the Huynh-Feldt *ε* correction. Paired *t*-tests were applied as post hoc analyses. All analyses were conducted with SPSS^®^ system for Windows, version 22.0.

## Results

### Visuomotor performance

**Fig 2:**
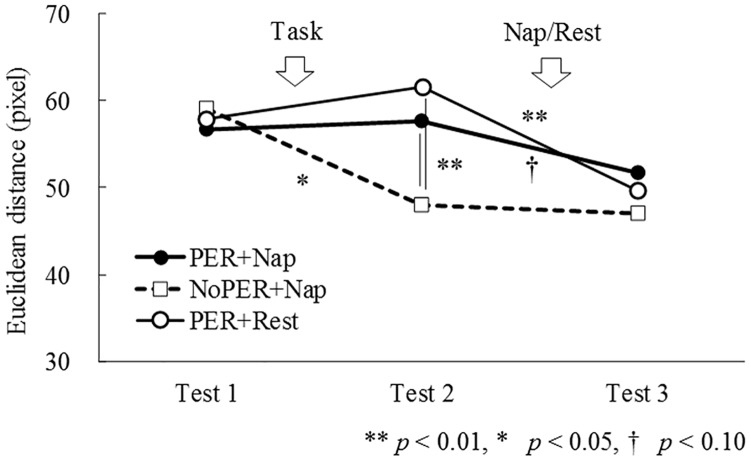
The distances between the target and the point where the mouse cursor. PER+Nap: perturbation and nap condition, NoPER+Nap condition: no-perturbation and nap, PER+Rest: perturbation and rest condition.

The main effect of Condition (*F*(2, 34) = 3.41, *ε* = 1.00 *p* < 0.05) and Time (*F*(2, 34) = 6.55, *ε* = 0.90 *p* < 0.01) and their interaction (*F*(4, 68) = 4.68, *ε* = 0.93 *p* < 0.01) were significant. As shown in Fig 2, visuomotor performance improved by practice (*t*(17) = 2.65, *p* < 0.05; the difference between Test 1 and 2) in the NoPER+Nap condition, whereas performance did not improve after the afternoon nap (*t*(17) = 0.39, *p* = 0.70; the difference between Test 2 and 3). Moreover, visuomotor performance did not change as a result of practice either in the PER+Nap, or in the PER+Rest conditions (*t*(17) = 0.24, *p* = 0.82; and *t*(17) = 0.94, *p* = 0.36 respectively; the difference between Test 1 and 2), whereas performance improved after the nap, and after the rest (*t*(17) = 1.76, *p* < 0.10; and *t*(17) = 4.59, *p* < 0.01 respectively; the difference between Test 2 and 3). Also, performance was significantly better in Test 2 in the NoPER+Nap condition compared to other conditions (*t*(17) = 2.97, *p* < 0.01 for the PER+Nap condition; *t*(17) = 4.27, *p* < 0.01 for the PER+Rest condition). These results indicate that (1) visuomotor performance during practice improved only in the no perturbation task, and that (2) improvements in visuomotor skills after the perturbation task was observed after both the nap and the rest periods.

### Flow and mood during the task

Flow experience scores during the task were grouped and averaged for each of three factors: “confidence,” “challenge” and “immersion,” and compared among conditions. The results indicated no significant differences among conditions as shown in Table 1 (*F*(2, 34) = 2.66, *ε* = 0.65, *p* = 0.11 for confidence; *F*(2, 34) = 0.39, *ε* = 1.00, *p* = 0.68 for challenge; *F*(2, 34) = 0.49, *ε* = 1.00, *p* = 0.62 for immersion). Furthermore, there were no significant differences in the mood among the conditions (*F*(2, 34) = 2.18, *ε* = 1.00, *p* = 0.13 for anxious, *F*(2, 34) = 0.21, *ε* = 1.00 *p* = 0.81 for sleepiness, *F*(2, 34) = 0.31, *ε* = 0.92, *p* = 0.96 for fatigue, *F*(2, 34) = 0.43, *ε* = 0.62, *p* = 0.56 for apathetic/vigorous, *F*(2, 34) = 1.05, *ε* = 0.79 *p* = 0.35 for confusion, *F*(2, 34) = 2.47, *ε* = 0.75, *p* = 0.12 for anger, *F*(2, 34) = 2.36, *ε* = 0.84, *p* = 0.12 for sadness). These results suggest that the visuomotor perturbation neither affected the flow experience nor the mood status.

**Table 1:** Scores of flow experience and moods during the task.

		PER+Nap	PER+Rest	NoPER+Nap	*p*
Flow	confidence	43.4 (5.19)	38.3 (3.74)	32.9 (4.67)	*n*.*s*.
	challenge	55.0 (5.58)	58.7 (3.80)	53.9 (5.49)	*n*.*s*.
	immersion	44.5 (4.77)	40.7 (3.33)	44.9 (4.63)	*n*.*s*.
Moods	anxiety	18.5 (6.03)	20.6 (4.84)	32.5 (7.21)	*n*.*s*.
	sad	12.7 (4.17)	14.6 (4.37)	24.7 (6.50)	*n*.*s*.
	anger	6.6 (2.01)	8.2 (2.56)	16.0 (4.88)	*n*.*s*.
	apathetic/vigorous	37.1 (5.16)	36.0 (4.11)	32.1 (5.07)	*n*.*s*.
	fatigue	50.4 (6.12)	49.5 (6.40)	51.0 (5.99)	*n*.*s*.
	confused	24.5 (6.07)	21.9 (4.88)	31.0 (6.61)	*n*.*s*.
	sleepiness	62.4 (5.45)	64.1 (4.97)	60.0 (6.20)	*n*.*s*.

The parentheses show standard errors.

### Psychomotor vigilance task

Median reaction times for the PVT indicated no significant differences in the main effect of condition (*F*(2, 34) = 0.69, *ε* = 0.93 *p* = 0.58) and time (*F*(2, 34) = 2.13, *ε* = 1.00 *p* = 0.14), or their interaction (*F*(4, 68) = 0.78, *ε* = 1.00 *p* = 0.54). These findings suggest that visuomotor perturbation did not affect the arousal level as measured by the PVT (Table 2).

**Table 2:** Median of the reaction time in the psychomotor vigilance test.

	Test 1	Test 2	Test 3	*p*
PER+Nap	313.0 (8.90)	317.7 (7.38)	307.4 (6.49)	*n*.*s*.
PER+Rest	312.8 (8.29)	320.8 (9.25)	310.9 (7.81)	*n*.*s*.
NoPER+Nap	311.2 (8.25)	310.0 (7.40)	304.8 (4.88)	*n*.*s*.

The parentheses show standard errors.

### Sleep stages

There were no significant differences in sleep stages between PER+Nap and NoPER+Nap conditions (Table 3). This finding suggests that visuomotor perturbation did not affect the quality of sleep after the task.

**Table 3:** Sleep architecture in the PER+Nap and NoPER+Nap conditions.

	Wake	1	2	3	4
PER + Nap	8.4 (3.68)	22.7 (2.77)	34.8 (3.19)	5.5 (2.09)	0.2 (0.17)
NoPER + Nap	7.9 (3.50)	24.5 (3.25)	37.1 (3.88)	4.6 (2.17)	0.3 (0.28)
*p*	*n*.*s*.	*n*.*s*.	*n*.*s*.	*n*.*s*.	*n*.*s*.
	REM	SWS	MT		
PER + Nap	11.6 (2.67)	5.6 (2.23)	1.0 (0.22)		
NoPER + Nap	11.1 (1.70)	4.8 (2.42)	0.8 (0.28)		
*p*	*n*.*s*.	*n*.*s*.	*n*.*s*.		

Means and SEs (in parentheses) are shown for time (in minutes) spent awake (Wake), in NonREM sleep stages 1, 2, 3 and 4, in REM (rapid eye movement) sleep, SWS (slow wave sleep), and for MT (movement time).

## Discussion

Results indicated that (1) the flow experience did not occur in the visuomotor task used in this study, which matched levels of skills and challenge, (2) improvements in visuomotor skills occurred regardless of the task perturbation, (3) improvements in visuomotor skills occurred unrelated to the flow or mood states, and (4) improvements in visuomotor performance were unrelated to sleep. These findings suggest that improvements in visuomotor skills resulting from the fit between the internal skill and the environment, as well as the development of new skills, are independent of perturbations from axis rotation, and are also independent of mood, including the flow experience. Results also indicated that elapsed time rather than sleep was related to visuomotor skills acquisition.

Contrary to the hypotheses of this study, we did not find any significant differences in flow and mood status among the conditions. This suggests that visuomotor perturbation has no influence on subjective experiences including the flow experience. According to the flow theory, the flow occurs even under low challenges, when task difficulty and skill level are matching [1, 2]. However, this contention was not supported by this study. Moreover, performance improvements observed in Test 3 suggest that the flow experience might be independent of the acquisition of skills, at least in visuomotor rotation tasks.

Previous studies have attempted to apply the flow theory in applied settings, such as sports and education. These studies have reported that people with a higher level of motor skills requiring implicit learning, such as racing car drivers [36], soccer players [6], and piano players [3] experience the flow during these activities. However, we did not manage to induce the flow experience in this study by using a simple visuomoter task under an experimental situation. As previous studies have demonstrated, the flow experience can more easily occur in applied situations than in experimental settings, such as those of the present study. In addition, flow experience is likely to be induced when people engage in more complicated task, i.e., when people find organized complexity in a task [2] than when engaging in simple tasks, such as that in this experiment.

Feedback mechanisms could provide another possible explanation of the non-significant flow experience found in this study: a person’s performance in the present task might not have induced sufficient sense of achievement and reward to induce the flow, because feedback was weak compared to situations examined in previous research. In sports settings, for instance, a person can get clear feedback regarding activities and is able to know how well the task was performed [1]. Feedback is a critical aspect of the reward system inducing the flow experience [1] [37]. Brain imaging studies using positron emission tomography (PET) have shown that the dopaminergic function in the striatum is related to individual differences in the flow experience (i.e., flow proneness) [37]. Feedback on performance is also related to the reward system, and therefore the flow experience could result from the following process: (1) Perception of clear feedback about performance, (2) activation of the dopaminergic system in the brain and then (3) generating subjective experiences including the flow. In the present study the lack of the first process, i.e., feedback on performance caused by an obscure rule for judging success might have resulted in a less intense flow experience during the visuomotor task, which could have resulted in the non-significant difference between conditions in this study. It is suggested that these possibility should be investigated in future studies.

The important finding of the present study was that the effect of a nap on visuomotor skill acquisition was no different from that of resting (see Test 3 in Fig 2). Moreover, performance in NoPER+Nap condition failed to improve in Test 3 compared to Test 2, which also suggested that the nap had no skill enhancing effects (there is a floor effect in skill improvement, or participants’ adaptation to the task might be an alternative explanations of the missing nap effect). Thus, the results of the study suggest that visuomotor skill acquisition did not depend on sleep, but rather on elapsed time. This finding is consistent with that of previous studies [21–25] and reinforces the assumption that sleep is irrelevant to improving implicit learning tasks including visuomotor learning [12]. We have reported previously that contextual learning, an example of implicit learning, is not enhanced by a 20 min afternoon nap taken after the task [38]. Taking these findings into consideration, it is suggested that implicit skill learning, including visuomotor and contextual skills, is not related to sleep itself, but rather to the elapsed time.

The effect of the perturbation on visuomotor performance was observed in Test 2 (see Fig 2), because task performance improved in the NoPER condition than in the PER conditions. This difference, however, disappeared in Test 3, which showed no significant differences among the three conditions. These results suggest that performance improvements during the PER conditions in Test 3 could have been caused by unmasking the aftereffects of perturbation. In the PER conditions, participants finished their practice sessions with approximately 60 degrees of axis rotation on average. Then, participants manipulated the mouse with zero degrees axis rotation (the normal mouse axis) in the test session that was conducted immediately after perturbation trials, which might have resulted in inertia, or aftereffects, following internal skill fitting [39]. The participants had to readapt to zero degrees, but they might not have adapted to this sudden change quickly, which could have resulted in aftereffects of axis perturbation. It is known that aftereffects of using a different internal skill (i.e., performance deterioration immediately after executing a different axis rotation task) disappear with elapsed time [40].

Participants in the axis rotation conditions had to develop a new internal skill for adapting to new combinations of visuomotor input, because new internal skills must be used to convert visual information to motor action when executing a visuomotor task. Additionally, participants have to use basic skills for executing the task, which they already possessed for controlling the mouse. The newly acquired skills and basic skills might independently contributed to mouse manipulation. The newly acquired skill for task rotation might have caused aftereffects and masked the basic skills improvement in Test 2 under perturbation conditions. In fact, increased performance improvements were observed in Test 3 compared to Test 2 (see Fig 2), suggesting that aftereffects could have disappeared with elapsed time, regardless of sleep or rest conditions.

A major limitation of this study, however, was there was no NoPER+Rest condition to control for the NoPER+Nap condition. As a result, the effect of the nap on the acquisition of visuomotor skills could not be precisely identified, although previous studies have indicated that the effect of this control condition is negligible [21–25]. Moreover, there is also the possibility that the visuomotor task was not appropriate for inducing the flow experience, because the perturbation to the control task difficulty might have induced an attentional interference or distraction. The distraction might have impeded participants’ concentration on the task and prevented the flow experience. Unfortunately, in the present study we did not measure the degree of subjective distraction in executing the perturbation task, or subjective task difficulty. It is suggested that this issue should also be considered in future studies.

In conclusion, (1) the flow experience does not occur when fitting skills levels and challenges, (2) flow experience is independent of learning visuomotor skills of using a mouse and (3) sleep is unrelated to visuomotor skill acquisition. It is suggested that these findings make an important contribution to discussions on the relationship between the flow experience and implicit learning.
